# Comparison of cyclophosphamide and calcineurin inhibitors for idiopathic membranous nephropathy: A protocol for systematic review and meta-analysis

**DOI:** 10.1097/MD.0000000000028891

**Published:** 2022-02-25

**Authors:** Kang Li, Yanqiu Yu, Yuan Gao, Junjie Gao

**Affiliations:** Department of Nephrology, Cangzhou Central Hospital, Hebei, China.

**Keywords:** calcineurin inhibitor, cyclophosphamide, idiopathic membranous nephropathy, meta-analysis

## Abstract

**Background:**

Idiopathic membranous nephropathy (IMN) is one of the leading causes of nephrotic syndrome in adults. We performed a protocol for systematic review and meta-analysis to compare the efficacy and safety of cyclophosphamide (CTX) with calcineurin inhibitors (CNIs) in the treatment of IMN.

**Methods:**

PubMed, EMBASE, Cochrane Central Register of Controlled Trials, and 3 Chinese databases (WanFang Data, Chongqing VIP and China National Knowledge Infrastructure) were searched from inception through January 2022 to identify randomized controlled trials that compared CTX with CNIs for patients with IMN. Systematic review and meta-analysis of the data will be performed in RevMan software (version 5.3) according to the preferred reporting items of systematic reviews and meta-analysis guidelines. Two authors independently performed the literature searching, data extraction, and quality evaluation. Risk of bias was assessed using the Cochrane Risk of Bias Tool for randomized controlled trials.

**Results:**

The results will be submitted to a peer-reviewed journal once completed.

**Conclusion:**

The conclusion of our research will provide evidence to help physicians to decide between CTX and CNIs therapy regimens for IMN patients.

**Open Science Framework registration number:**

10.17605/OSF.IO/G584K

## 1 Introduction

Membranous nephropathy is an autoimmune glomerular disease in which immunoglobulins and complement proteins form immune complexes that start depositing on the subepithelial layer of the glomerular capillaries to form lesions.^[1,2]^ Membranous nephropathy is most usually associated with increased proteinuria and approximately 80% of patients have nephrotic syndrome. Globally, the annual incidence of membranous nephropathy is 1.2/100,000 in adults and 0.1/100,000 in children.^[3]^

Historically, membranous nephropathy was believed to result from trapping of preformed circulatory soluble immune complexes without any immunogenic involvement of the glomerulus. However, in late 1970s, it became evident that the reaction of free antibodies with glomerular antigens forms the immune complexes that result in their in situ deposition.^[4]^ Later studies identified that autoimmunity develops mainly (approximately 85%) against the M-type phospholipase A_2_ receptor and to a lesser extent (3%–5%) against the thrombospondin domain containing protein 7A or other unidentified antigens.^[5]^

Approximately 80% of the cases are classified as primary or idiopathic membranous nephropathy (IMN) in which the autoimmunity cause remains unknown. IMN is a chronic disease with a clinical course that is difficult to predict because many patients achieve spontaneous and have good prognosis, but others progress to end stage renal disease.^[6,7]^ Approximately 40% to 50% of the patients achieve complete or partial remission of proteinuria spontaneously usually with renal stability whereas a gradual progression to end stage renal disease is observed in 5 to 15 years in rest of the patients.^[8]^

Alternating monthly cycles of alkylating agents [especially cyclophosphamide (CTX)] and corticosteroids has been proven to be more effective than corticosteroids alone in inducing remissions and preserving renal function.^[9,10]^ However, CTX is accompanied by frequent adverse drug reactions. For patients who cannot tolerate or refuse to CTX treatment, calcineurin inhibitors (CNIs), including cyclosporine (CSA) and tacrolimus (TAC), are suggested to be promising alternatives.^[11,12]^ However, it is difficult for physicians to decide between CTX and CNIs therapy regimens for IMN patients. Therefore, we performed a protocol for systematic review and meta-analysis to compare the efficacy and safety of CTX with CNIs in the treatment of IMN.

## 2 Methods

### 2.1 Study registration

This systematic review protocol has been registered in the Open Science Framework registries (registration number: 10.17605/OSF.IO/G584K). We will follow recommendations outlined in the Cochrane handbook of systematic review of interventions and the preferred reporting items for systematic reviews and meta-analysis protocol statement guidelines.^[13]^ If amendments are needed, we will update our protocol to include any changes in the whole process of research. Since this study is on the basis of published studies, ethical approval is not required.

### 2.2 Search strategy

PubMed, EMBASE, Cochrane Central Register of Controlled Trials, and 3 Chinese databases (WanFang Data, Chongqing VIP, and China National Knowledge Infrastructure) were searched from inception through January 2022 to identify randomized controlled trials that compared CTX with CNIs for patients with IMN. Combinations of the following search terms were used: IMN, idiopathic membranous glomerulonephritis, calcineurin phosphatase inhibitor, CSA, TAC, FK506, alkylating agents, modified ponticelli regimen, CTX, cytotoxic drug, randomized controlled trial, and placebo controlled trial. Reference lists of included papers and previous reviews were hand-searched for additional studies.

### 2.3 Inclusion criteria and study selection

RCTs with the following criteria were included: the study population was adults with biopsy-proven IMN, the study compared CTX plus corticosteroids with CNIs (CSA or TAC) plus corticosteroids, and the study reported at least one of the necessary outcomes regarding efficacy and safety. The following indexes were used as efficacy outcomes: total remission rate, improvement of serum albumin, and reduction of proteinuria (measured as g/24 h). The relapse rate was used as an indirect index to evaluate efficacy outcomes. While the following indexes were used as safety outcomes: final serum creatinine, incidence of adverse drug reactions, and dropout rate due to adverse drug reactions. The article languages were limited to Chinese and English. Studies that recruited pediatric patients and studies that focused on secondary membranous nephropathy were excluded. Study eligibility was independently determined by 2 investigators. Disagreements were resolved through discussion among the authors.

### 2.4 Data extraction

Data extraction was performed by 2 independent investigators according to a predesigned review form. Disagreements were resolved through discussion among all authors. Assessed variables related to the studies were extracted, including the characteristics of the studies (the authors, publication year, and inclusion criteria), the characteristics of the populations (numbers of patients, age, and gender), the intervention regimens (dose, administration route, and treatment duration), the baseline laboratory test values (proteinuria and serum albumin), and the follow-up information (follow-up length and lost to follow-up rate).

### 2.5 Quality assessment

Two authors independently assessed the quality of the included studies. Disagreements were resolved through discussion among all authors. The quality of included studies was assessed using the Cochrane risk of bias assessment tool^[14]^ for the following 6 aspects: random sequence generation, allocation concealment, blinding of participants and investigators, blinding of outcome assessors, incomplete outcome data, and selective reporting.

### 2.6 Statistical analysis

The meta-analyses were performed using RevMan (IBM) software (version 5.3). Dichotomous and continuous outcomes were expressed as the odds ratio and mean difference with 95% confidence interval, respectively. Heterogeneity was assessed by χ^2^ test (*P* < .1 indicating statistical significance) and I^2^ test. A random-effect model was used for data analysis (*P* < .05 indicating statistical significance). Considering that different administration routes of CTX may result in different conclusions, an additional subgroup analysis based on oral CTX and intravenous CTX was performed. A sensitivity analysis was also performed by changing the analysis method from random-effect model to fixed-effect model and by excluding studies with uncertain eligibility. Publication bias was identified by funnel plots if there was an adequate number of included studies (ie, at least 10 studies).

## 3 Discussion

IMN is one of the leading causes of nephrotic syndrome in adults. Current guidelines recommend steroid plus CTX as the initial therapy for patients with IMN.^[15]^ Although this combined regimen has demonstrated a good effectiveness in remission, it is associated with severe side-effects. Considering the advanced age of the majority of IMN patients, numerous adverse effects of these aggressive regimen are an important concern. As one of the CNIs, a RCT study showed that a majority of patients who received TAC monotherapy experienced remission with a significant reduction in the risk for deteriorating renal function in IMN.^[16]^ Besides, some previously reported studies indicated that the combination of TAC and steroid was as effective as the combination of CTX and steroid for IMN patients in achieving remission of severe proteinuria, and a higher incidence of remission was found in the short term in combination of TAC and steroid.^[17,18]^ However, most studies had a small sample size and short-term follow up, and few studies have been conducted to compare the safety profiles in 2 treatment protocols. Thus, we conducted this review to provide better evidence and guidance for clinical decision-making. We plan to publish this review within 1 year after the publication of the protocol, and we will update it every 3 years.

## Author contributions

Junjie Gao planned the study design. Yanqiu Yu reviewed the study protocol. Yuan Gao will recruit participants and collect data. Kang Li wrote the manuscript. All of the authors have read and contributed to the submitted manuscript.

**Conceptualization:** Yuan Gao.

**Data curation:** Yuan Gao.

**Investigation:** Yanqiu Yu, Yuan Gao.

**Writing – original draft:** Kang Li.

**Writing – review & editing:** Junjie Gao.
